# Case report: A preterm infant with rubinstein-taybi syndrome and Marmorata telangiectatica harboring a frameshift mutation in the CREBBP gene

**DOI:** 10.3389/fped.2023.1059658

**Published:** 2023-03-03

**Authors:** Yang Yang, Jing Xiao, Yuanyuan Ye, Jianwen Xiang, Zhu Wang, Jia Chen

**Affiliations:** Neonatal Department, Guangdong Women and Children Hospital, Guangzhou, China

**Keywords:** rubinstein-taybi syndrome, cutis marmorata telangiectatica congenita, CREBBP, case report, preterm

## Abstract

Rubinstein-Taybi syndrome (RSTS) is a rare autosomal dominantly inherited disease characterized by slow mental and physical growth, skeletal abnormalities (broad thumbs and big toes), and dysmorphic facial features. RSTS is associated with *de novo* variants of the epigenetic-associated gene CREBBP. RSTS is primarily diagnosed based on clinical manifestations and genetic testing. Cutis marmorata telangiectatica congenita (CMTC) is a rare, congenital, and typically benign vascular anomaly of unknown etiology; it is described as persistent reticulated marbled erythema. The diagnosis of CMTC is largely based on clinical features, and *GNA11* mutations are associated with CMTC. In this case report, we describe the case of a preterm infant (boy) with RSTS and CMTC who had a novel frameshift mutation leading to a premature stop codon in the CREBBP gene. This study adds the novel mutation c.5837dupC to the known molecular spectrum of disease-causing *CREBBP* gene mutations.

## Introduction

Rubinstein-Taybi syndrome (RSTS), a rare autosomal dominantly inherited disease, is characterized by slow mental and physical growth, skeletal abnormalities (broad thumbs and big toes), and dysmorphic facial features (1). Other manifestations of RSTS include heart defects, genitourinary and central nervous system malformations, skin anomalies, and an increased predisposition to cancer (2). RSTS is mainly caused by *de novo* variants in the epigenetic-associated gene *CREBBP* (3). Moreover, the diagnosis of RSTS mainly depends on clinical manifestations and genetic testing.

Cutis marmorata telangiectatica congenita (CMTC) is a rare, congenital, and benign vascular anomaly of unknown etiology (4). It is described as a persistent reticulated, marbled erythema that blanches with pressure and does not resolve with heating. As it affects capillaries and venules, it is characterized by a slow-flow vascular lesion (5). The diagnosis of CMTC is primarily based on clinical features. Recently, *GNA11* mutations have been reported to be associated with CMTC (6–8).

In the present study, we report the case of a preterm infant boy with RSTS and CMTC who presented with a novel frameshift mutation leading to a premature stop codon in the CREBBP gene. The child, with a typical facial appearance, experienced feeding difficulties, growth regression, and recurrent respiratory infections.

## Case report

**Figure 1:**
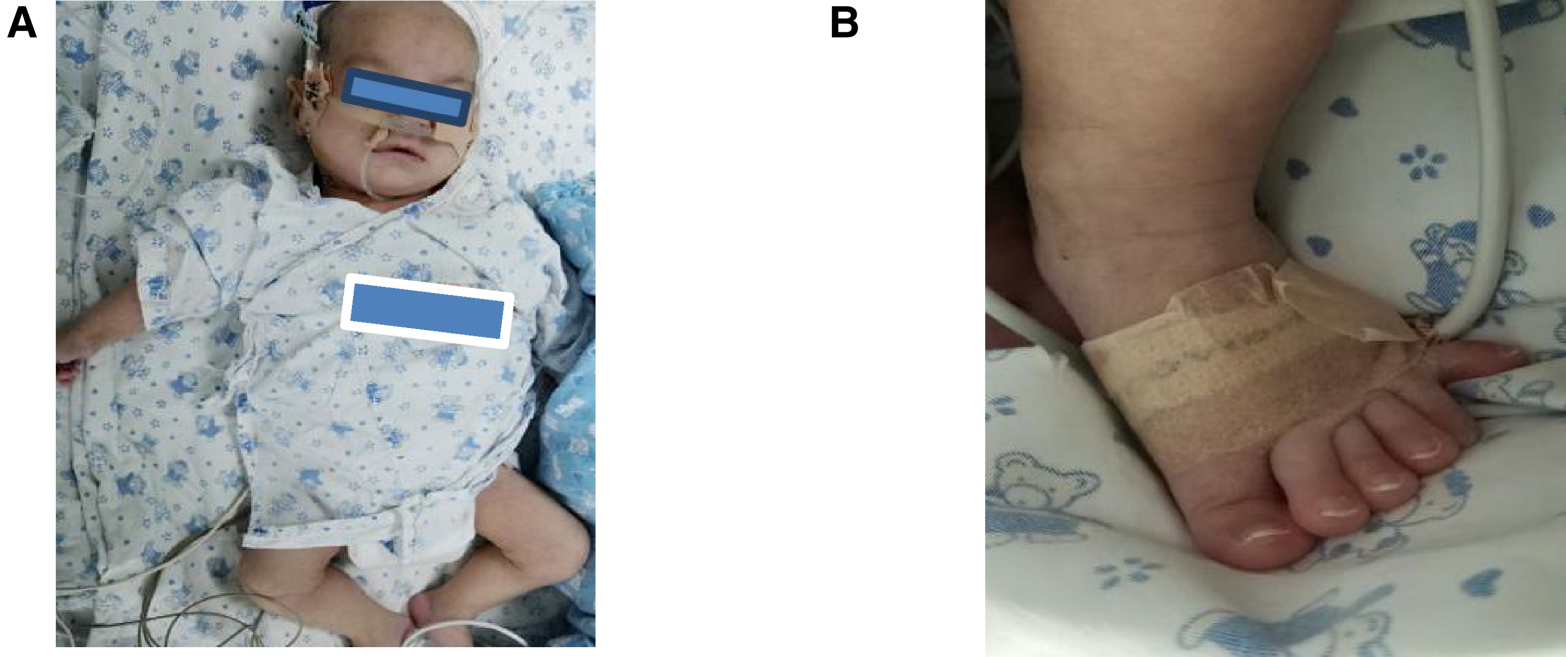
**(A)** A reticular erythematous path across the body, arched eyebrows, down-slanting palpebral fissures, a broad nasal bridge, and a triangular mouth. **(B)** The patient had polydactyly and broad big toes.

A boy, born at 34 weeks gestation, was transferred to our neonatal department after birth, requiring surfactant therapy and mechanical ventilation because of respiratory distress syndrome. The Apgar scores were 9–10–10. He weighed 2230 g (25th–50th percentile), was 43 cm long (10th–25th percentile), and his head circumference was 30 cm (10th–25th percentile). The patient presented with respiratory distress and polydactyly in both legs. In addition to the scalp, palm, sole, and mucous membranes, his entire body was covered with reticular marble-resembling flower-like macules (Figure 1). The skin changes did not disappear as the temperature in the environment increased. There was no family history of vascular malformation. No other congenital or neurological anomalies were observed. Repeat ultrasound examinations of the head and ophthalmological examinations were normal. Electroencephalography (EEG) and brain magnetic resonance imaging (MRI) revealed no abnormalities. Ear emission and auditory evoked potentials were normal, and echocardiography findings showed patent foramen ovale (PFO). Urinary ultrasound was normal, and thyroid function test results were all within normal limits. Due to respiratory distress and feeding difficulties, the patient stayed in the intensive care unit for approximately three months. Despite receiving antibiotics, respiratory support, and nasal feeding, his weight was 3800 g (<3rd percentile), his height was 53 cm (3rd–10th percentile), and his head circumference was 35 cm (<3rd percentile). In addition to microcephaly, he had arched eyebrows, down-slanting palpebral fissures, a broad nasal bridge, a triangular mouth, and broad big toes (Figure 1). At the age of four months, his Geisel score was equivalent to 4–8 weeks. In addition, the reticular marble-resembling flowers-like macules that covered his body didn't disappear.

During the most recent follow-up, at the time of writing this manuscript, the patient was two years old, could only speak a few words, and could not walk independently.

The baby covered with reticulated marbled erythema had dysmorphic facial features, broad toes, and mental and developmental delays. Our hypothesis is a diagnosis of RSTS with CMTC. However, genetic testing is required to confirm.

**Figure 2:**
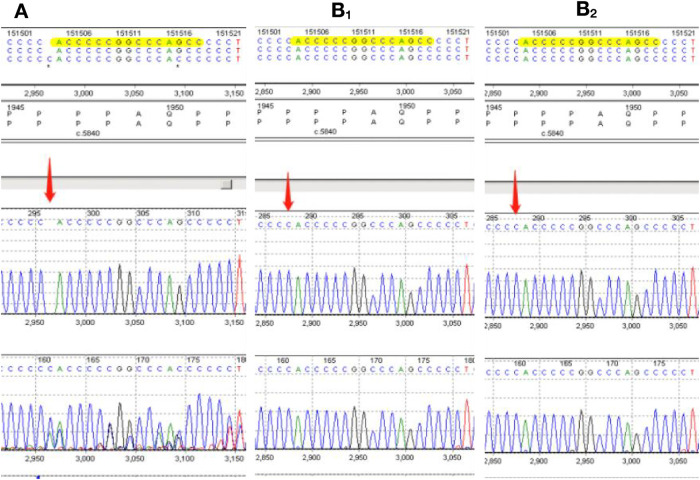
**(A**) sequence analysis of the genomic DNA of our rubinstein-taybi patient. Direct analysis of the *CREBBP* sequence reveals the C.5837dupC(p.P1974Thrfs*19) mutation. The *CREBBP* sequences of the patient's parents are also displayed **(B_1,_ B_2_).**

When RSTS was suspected, the clinical findings were supplemented with *CREBBP* sequencing using next-generation genotyping techniques. Sequencing reactions were performed using the MiSeq Illumina sequencer, and data analysis was performed using the MiSeq Reporter. *CREBBP* sequencing revealed a C duplication at position c.5837 in the *CREBBP* gene (c.5837dupC), a frameshift mutation predicted to result in premature termination at the 1966th amino acid of the CREB-binding protein (p.Pro1947Thrfs*19). Neither parent had any abnormalities at this site (Figure 2). Therefore, RSTS was confirmed.

### Discussion

RSTS, first described in 1963 by Rubinstein and Taybi (9), is caused by *CREBBP* mutations (10). To date, more than 500 *CREBBP* gene variants have been described in The Human Gene Mutation Database (HGMD) (3, 11). CREB-binding protein (CBP), encoded by *CREBBP*, is a histone and acetyl/lysine-transcription co-activator with more than 400 described interaction partners that regulate the expression of numerous genes during development and postnatal life (12). In this study, we present the case of an infant who presented with microcephaly, arched eyebrows, down-slanting palpebral fissures, a broad nasal bridge, a triangular mouth, broad big toes, polydactyly, postnatal developmental delay, and cognitive impairment.

Menke-Hennekam syndrome is characterized by the same facial dysmorphia as RSTS and is accompanied by epilepsy and hearing impairment. In our case, the baby had a big hallux and normal EEG and hearing tests. Moreover, Treacher-Collins syndrome, which is associated with mutations in the *TCOF1*, *POLRIC*, and *POLRID* genes, is marked by microcephaly, an intellectual deficit, and other congenital malformations (13). The main distinguishing features of the three diseases are a grimacing smile and broad toes or fingers (14,15). Large toes or fingers are also a manifestation of Pfeiffer syndrome, which is related to the *FGFR1* or *FGFR2* genes and neural structural malformations (13, 16). Moreover, Comelia de Lange and Floating-Harbour syndromes are also defined by intellectual deficits and other anomalies similar to RSTS; however, both are characterized by skeletal dysplasias such as those of the ulna and skull (13). In the present case, the brain MRI was normal, further confirming RSTS with CMTC.

RSTS can be accompanied by skin anomalies, with seven cases reported to date (11, 17). However, all of these reports dealt with neural crest-derived tumors, keloid or hypertrophic scars, supernumerary nipples, ingrown toenails, paronychia, hypertrichosis, and glabellar hemangiomas (11, 17). The c.5837dupC (p.Pro1947Thrfs*19) mutation in *CREBBP* identified in this study has also been detected in both a five-year-old girl and a 12-year-old girl with RSTS, and one of these girls developed pilomatricomas. In our case, RSTS was accompanied by CMTC. Although all three cases (ours and the two girls') had the same mutation, the phenotypes differed. Therefore, the same mutation may contribute to the disease *via* multiple molecular pathways. Furthermore, some studies have suggested a link between CMTC and mutations in *GNA11* (5). However, in our case, CMTC was caused by a mutation in *CREBBP*.

The patient in our case study had polydactyly, similar to a previous report, and we discovered that these symptoms are extremely common in Chinese patients. However, this high incidence has rarely been reported in previous patients with RSTS (3). Moreover, studies addressing genotype-phenotype correlations do not detect statistically significant differences between RSTS cases with distinct mutations or between RSTS cases with and without mutations (18). However, our case report indicates that the mutations in the same gene may have different clinical manifestations in RSTS, warranting further investigation.

## Data Availability

The datasets presented in this study can be found in online repositories. The names of the repository/repositories and accession number(s) can be found in the article/Supplementary Material.
